# Chronic Lyme borreliosis associated with minimal change glomerular disease: a case report

**DOI:** 10.1186/s12882-017-0462-4

**Published:** 2017-02-06

**Authors:** N. Florens, S. Lemoine, F. Guebre-Egziabher, F. Valour, J. Kanitakis, M. Rabeyrin, L. Juillard

**Affiliations:** 1 Department of Nephrology, Dialysis and Hypertension, Edouard Herriot Hospital, Hospices Civils de Lyon, 5 Place d’Arsonval, 69437 Lyon, Cedex 03 France; 2 Université Claude Bernard Lyon 1, Villeurbanne, France; 3 INSERM U1060, CarMeN, Université Claude Bernard Lyon 1, Lyon, France; 4 Department of Infectious and Tropical Diseases, Hospices Civils de Lyon, Lyon, France; 5 Deparment of Dermatology, Edouard Herriot Hospital, Hospices Civils de Lyon, Lyon, France; 6 Department of Pathology, Edouard Herriot Hospital, Hospices Civils de Lyon, Lyon, France

**Keywords:** Lyme, Borreliosis, Minimal change disease, Chronic atrophic acrodematitis

## Abstract

**Background:**

There are only few cases of renal pathology induced by Lyme borreliosis in the literature, as this damage is rare and uncommon in humans. This patient is the first case of minimal change glomerular disease associated with chronic Lyme borreliosis.

**Case presentation:**

A 65-year-old Caucasian woman was admitted for an acute edematous syndrome related to a nephrotic syndrome. Clinical examination revealed violaceous skin lesions of the right calf and the gluteal region that occurred 2 years ago. Serological tests were positive for Lyme borreliosis and skin biopsy revealed lesions of chronic atrophic acrodermatitis. Renal biopsy showed minimal change glomerular disease. The skin lesions and the nephrotic syndrome resolved with a sequential treatment with first ceftriaxone and then corticosteroids.

**Conclusion:**

We report here the first case of minimal change disease associated with Lyme borreliosis. The pathogenesis of minimal change disease in the setting of Lyme disease is discussed but the association of Lyme and minimal change disease may imply a synergistic effect of phenotypic and bacterial factors. Regression of proteinuria after a sequential treatment with ceftriaxone and corticosteroids seems to strengthen this conceivable association.

## Background

Lyme disease-associated nephritis is rare in humans. The majority of reported cases are related to membranoproliferative glomerulonephritis (MPGN) caused by renal deposition of immune-complexes [1–4]. We report here a unique case of minimal change disease (MCD) associated with chronic Lyme borreliosis that resolved completely after sequential treatment with ceftriaxone and corticosteroids.

## Case presentation

### Clinical features

**Fig. 1:**
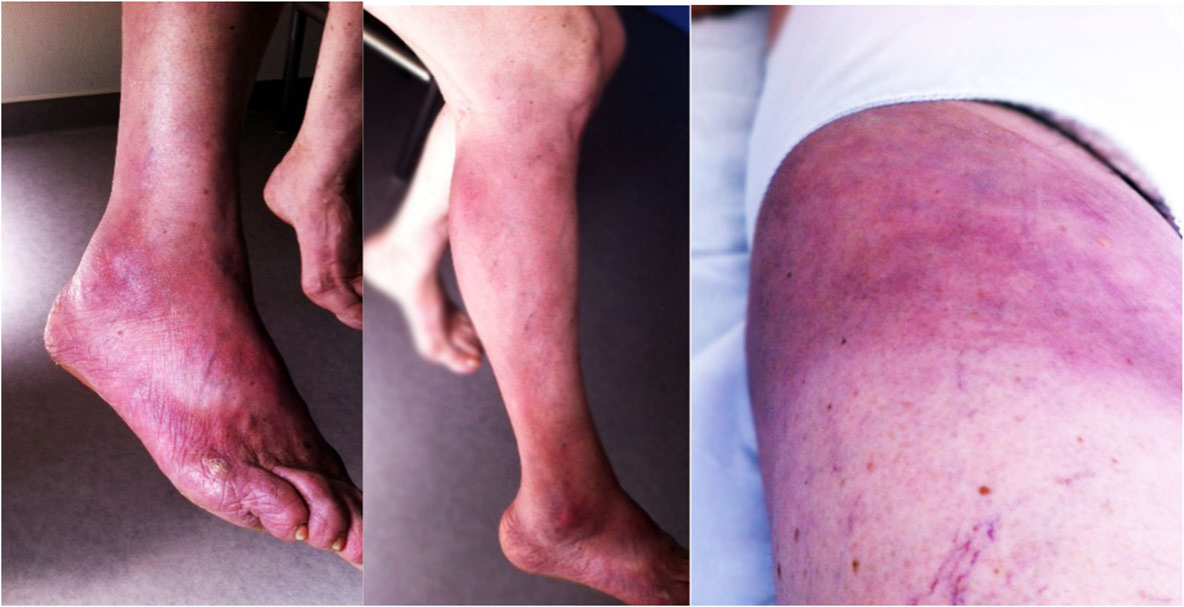
Clinical features of chronic atrophic acrodermatitis. Lesions started from the back of the foot and spread to the inguinal and gluteal regions. Areas of uninvolved skin areas are present between the lesions

A 65-year-old woman was admitted for an acute edematous syndrome associated with asthenia. The patient had a past medical history of hypertension, dyslipidemia, bilateral gonarthrosis and left coxarthrosis. On admission, physical examination revealed arterial hypertension (163/89 mmHg) without fever and a violaceous cutaneous rash of the right leg that had appeared 2 years before. It had started as a small painful round macule of the foot that progressively extended to the whole calf and the gluteal region (Fig. 1). Neurological examination revealed painful peripheral neuropathy on the right leg. Electrocardiogram was normal.

Laboratory data showed hypoalbuminemia (1,2 g/dl) associated with severe proteinuria (7,03 g/24 h) and a recent decrease of renal function (serum creatinin level increase from 0,84 to 1,3 mg/dl). Urinalysis showed cytolipiduria without hematuria or pyuria (11/mm3 and 22/mm3, respectively). Serological tests for viral infections (HBV, HCV, EBV, HIV), antinuclear and antineutrophil cytoplasmic antibodies were negative. Complement was normal. Cryoglobulin (monoclonal IgM-kappa and polyclonal IgG) was found. Serum protein electrophoresis revealed an oligoclonal increase of two IgG-lambda and one IgM-lambda condensations.

**Fig. 2:**
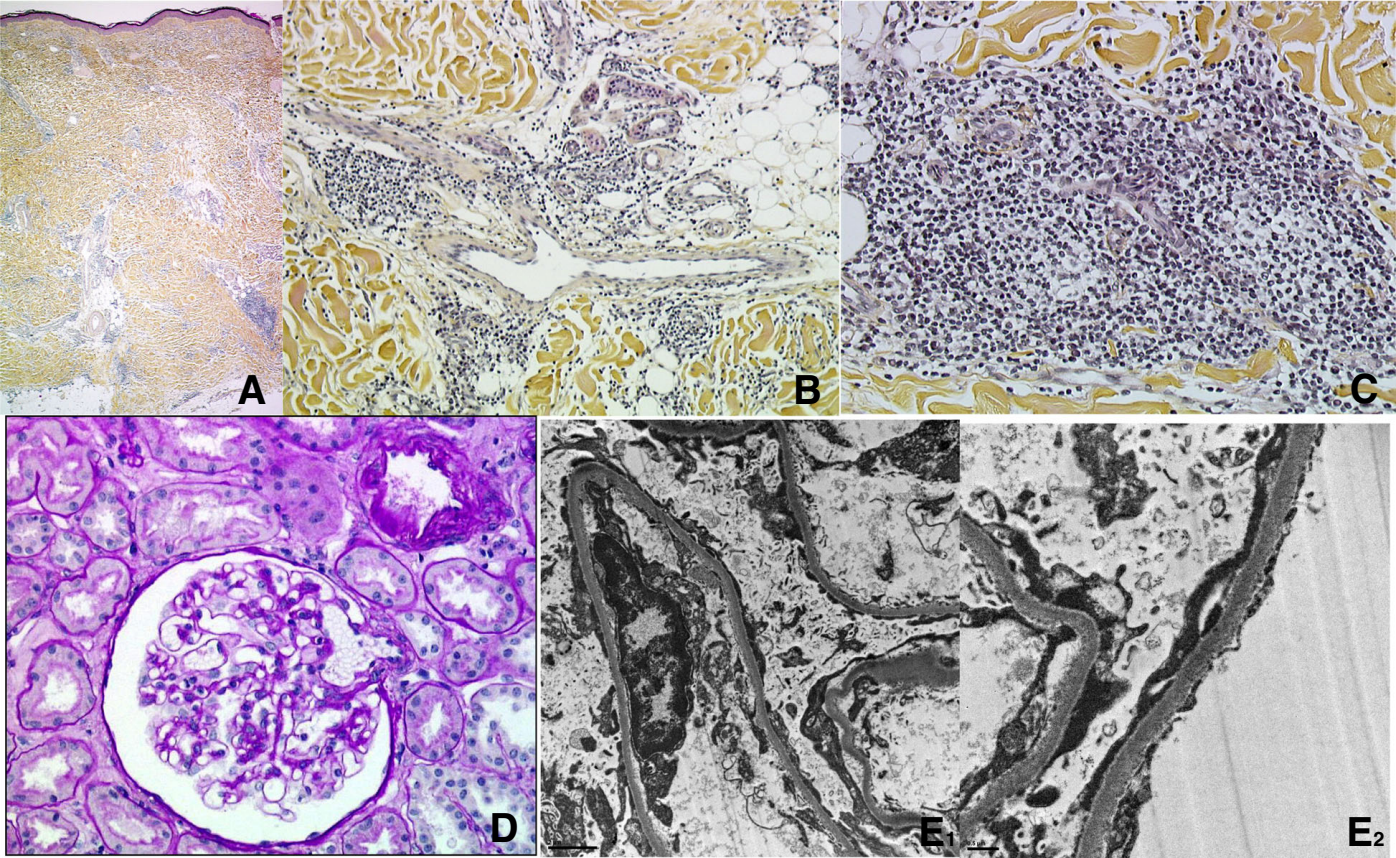
Biopsies features. Skin biopsy: **a** Scanning magnification (X25): a patchy perivascular and periadnexal cell infiltrate is present in the dermis (HES stain). **b** Medium-power magnification (X100): a mononuclear cell infiltrate is present in the deep dermis around dilated vessels and sweat glands (HES stain). **c** High-power magnification (X250): a dense lymphoplasmocytic perivascular infiltrate is present in the deep dermis (HES stain). Renal biopsy: **d** Optical microscopy (X200): normal glomerulus with no segmental lesion (PAS stain). **e**_**1**_ Transmission electron microscopy (X8000): podocyte effacement without deposits or double contour (Uranyl acetate and lead citrate stain). **e**_**2**_ Transmission electron microscopy (X20000): podocyte effacement without deposits or double contour (Uranyl acetate and lead citrate stain)

Histological examination of the skin biopsy showed a diffuse, rather dense dermal infiltration reaching the subcutaneous adipose tissue. It consisted predominantly of CD3+/CD5+ T-lymphocytes, admixed with numerous CD79a+/CD138+ polyclonal plasma cells (expressing in comparable amounts kappa and lambda light chains) and fewer CD20+ B-cells suggestive with chronic atrophic acrodermatitis (Fig. 2).

Serological tests for Lyme disease confirmed the diagnosis of chronic borreliosis: the enzyme-linked immunosorbent assay was positive for IgG (titer > 200 U/mL, normal value < 6) and IgM (Enzygnost® Lyme link VLsE/IgG and Borreliosis/IgM used on the BEPIII® system, Siemens Healthcare Diagnostic Products GmbH, Marburg, Germany), and was confirmed by immunoblotting. After further questioning, the patient recalled a tick-bite 9 years before on this leg, but neither migratory erythema nor arthritis.

A renal biopsy disclosed chronic vascular lesions and normal glomeruli devoid of immune deposits (Fig. 2). Transmission electron microscopy showed a diffuse podocyte effacement without any evidence of deposits (Fig. 2). Specific polymerase chain reaction (PCR) by LightMix Kit for the detection of Borrelia spp. (TIB MOLBIOL, ROCHE, Berlin, Germany) performed on DNA extracted from both skin and kidney biopsies was negative.

A whole-body scan showed no evidence of lymphoma. Renal ultrasound and doppler examinations were normal. The patient was not taking any medication that could induce the MCD.

### Evolution with treatment

Proteinuria slightly decreased after administration of an angiotensin-converting enzyme inhibitor (ACEi) at d8. After serological confirmation of Lyme borreliosis, ceftriaxone was initiated (2 g/day by subcutaneous injections starting on day 21 after diagnosis, d21). One week later (i.e. on d28), corticosteroids (CS) were added (70 mg prednisolone, i.e. 1 mg/kg). To prevent an infection flare, CS administration was delayed.

**Fig. 3:**
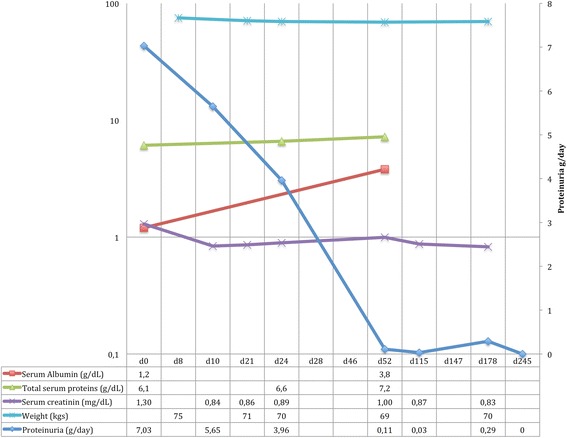
Evolution of biologic and clinical parameters in response of therapeutic interventions. Left vertical axis: log scale. **d0:** diagnosis. **d8:** ACEi introduction. **d21:** Ceftriaxone introduction. **d28:** Corticosteroid (CS) introduction. **d46:** end of ceftriaxone therapy. **d52:** Start of the CS Tapering, 2.5 mg per week. **d115:** ceftriaxone re-introduction, CS 47.5 mg. **d147:** end of second ceftriaxone therapy. **d178:** CS 17.5 mg/day, tapering of 2.5 mg/15 days until 10 mg then 1 mg every month. d245: CS 9 mg. Note : conversion factor units : Serum creatinine in mg/dL to μmol/L x 88,4

At d24, i.e. 72 h after initiation of ceftriaxone therapy, proteinuria decreased again (3.96 vs 7.03 before treatment and vs 5.96 g/24 h after ACEi introduction) and hypertension and the edematous syndrome improved. At d48, the first antibiotherapy course was discontinued. At d52, proteinuria was insignificant (0.11 g/day) and CS were rapidly tapered (by 2.5 mg/week). At d115, while CS dosage was 47.5 mg, proteinuria was still insignificant (0.03 g/day) but the neuropathic pain of the right foot persisted. Electroneuromyographic and lumbosacral remnography remained normal. Ceftriaxone therapy was again started in association with pregabalin until d147 and resulted in complete regression of the leg lesions and pain. At d178, with 17.5 mg of CS, proteinuria remained insignificant (0,29 g/day) and CS were further tapered (2.5 mg every 15 days until 10 mg and then 1 mg every month). At d245, with 9 mg CS, there was a complete remission of the nephrotic syndrome (Fig. 3). CS were totally stopped at d350 and after a 3-years follow-up, proteinuria remained insignificant.

Serological tests after 6 months and a 3-years follow-up were still positive for IgG and became negative for IgM.

## Discussion

Lyme disease is a tick-borne infection caused by *Borrelia burgdorferi*, and can present with various clinical manifestations due to the ability of spirochetes to affect many tissues [5]. Our patient seems to be the first case of MCD associated with Lyme disease. This calls for several comments.

*Borrelia burgdorferi* is known to induce Lyme nephritis especially in dogs. In most cases, pathological findings are membranoproliferative glomerulonephritis (MPGN) with Lyme-specific antigen-antibody complex deposition on the basal glomerular membrane [6–8]. In humans, we found four MPGN [1–4] one crescentic and IgA-deposit nephropathy [2] and a membranous nephropathy [9] in the setting of Lyme disease.

Lyme disease-associated nephropathy is rare and its pathogenesis still unclear. Role of immunomodulatory phenomena such as the deposition of immune complexes mediated by Lyme infection can be involved [6–8]. Bacterial lipopolysaccharides (LPS) can favor the development of MCD via disorganization of the podocyte cytoskeleton. This phenomenon is explained by the upregulation of B7-1, a costimulating factor responsible for glomerular permeability, induced by LPS biding with toll-like receptor 4 (TLR4) [10]. Interestingly, in Lyme disease, there is an upregulation of the expression of B7-1 and B7-2 [11]. Some lipoproteins present on the surface of the bacteria can bind and activate TLR 1, 2 and 4 [12]. Therefore, MCD in our case may be due to a strong upregulation in podocytes of B7-1 after the binding of lipoprotein from the surface of the bacteria with TLR4.

The decrease of proteinuria after ceftriaxone therapy observed in our case suggests a link between MCD and Lyme disease. As described above, T-cells, podocytes and bacterial wall antigens could be involved. Nevertheless, this phenomenon has not been so far described in other cases of chronic Lyme disease and despite ceftriaxone, CS, ACEi and low-sodium diet prescribed during the hospitalization may themselves explain a decrease of the proteinuria [13].

The co-existence of cryoglobulin and oligoclonal proliferation of immunoglobulins on plasma electrophoresis in the setting of MCD rendered important the exclusion of a lymphoma. However, the results of the initial workup were reassuring, and 3-years follow-up did not disclose any evidence for it. Furthermore, drug-associated MCD was excluded only with questioning, so that an omission cannot be excluded definitely.

On the other hand, the diagnostic value of PCR in Lyme disease remains unclear as it is used mainly for research [5]. Besides, PCR was also negative in another case report [1], and was not mentioned in other studies [2–4, 9]. Moreover, in dogs, immunohistochemistry assay did not show any evidence of renal invasion of Borrelia in kidney tissues in dogs with suspected Lyme nephritis [8], as well as results of PCR assays were only positive for one biopsy on 4 dogs with a positive or equivocal status for Lyme borreliosis [6].

Concerning treatment, our strategy introduced an unsolved question: was ceftriaxone alone able to treat MCD in our case? Besides ceftriaxone, the patient was also treated by corticosteroids, the reference treatment for MCD. Moreover, we added ACEi that had an effect on the decrease of the proteinuria. This association allowed a complete remission of MCD (negative proteinuria at d52 and after a 3-years follow up). In previous studies about infection-related MPGN treatment, antibiotics were first started, corticosteroids delayed and then tapered [14]. Successful treatment resulted from the synergistic effect of antibiotics on bacterial inoculum and steroids on immune system.

## Conclusion

Renal damage is rare in human Lyme disease and mostly corresponds to MGPN. We reported here the first case of MCD associated with Lyme disease. The involvement of podocytes, T-cell mediated immune response and bacterial wall antigens can be considered. It can be speculated that some idiopathic MCD cases may be due to latent Lyme (or other infectious diseases) involving T-cell proliferation and podocyte dysfunction. The association of Lyme disease and MCD implies a synergistic effect of phenotypic and bacterial factors.

## Abbreviations

ACEi: Angiotensin-converting enzyme inhibitor
CS: Corticosteroids
MCD: Minimal change disease
MGPN: Membranoproliferative glomerulonephritis
